# Coarse-Grained Models Reveal Functional Dynamics - I. Elastic Network Models – Theories, Comparisons and Perspectives

**DOI:** 10.4137/bbi.s460

**Published:** 2008-03-04

**Authors:** Lee-Wei Yang, Choon-Peng Chng

**Affiliations:** Institute of Molecular and Cellular Biosciences, University of Tokyo, Tokyo 113-0032, Japan

**Keywords:** normal mode analysis, potential surface, low-frequency motions, GNM, NMR, X-ray, B-factors

## Abstract

In this review, we summarize the progress on coarse-grained elastic network models (CG-ENMs) in the past decade. Theories were formulated to allow study of conformational dynamics in time/space frames of biological interest. Several highlighted models and their underlined hypotheses are introduced in physical depth. Important ENM offshoots, motivated to reproduce experimental data as well as to address the slow-mode-encoded configurational transitions, are also introduced. With the theoretical developments, computational cost is significantly reduced due to simplified potentials and coarse-grained schemes. Accumulating wealth of data suggest that ENMs agree equally well with experiment in describing equilibrium dynamics despite their distinct potentials and levels of coarse-graining. They however do differ in the slowest motional components that are essential to address large conformational changes of functional significance. The difference stems from the dissimilar curvatures of the harmonic energy wells described for each model. We also provide our views on the predictability of ‘open to close’ (open→close) transitions of biomolecules on the basis of conformational selection theory. Lastly, we address the limitations of the ENM formalism which are partially alleviated by the complementary CG-MD approach, to be introduced in the second paper of this two-part series.

## Introduction

Protein has a dynamic nature. Dynamics encoded in the evolutionally optimized (Leo-Macias et al. 2005) structures are coupled with catalytic chemistry in facilitating protein functions (Eisenmesser et al. 2002; Wolf-watz et al. 2004; Yang et al. 2005a). The ‘jiggling and wiggling’ atoms were understood in further depth when Koshland turned the ‘lock-and-key’ paradigm (Fischer, 1894) into an ‘induced-fit’ fever (Koshland, 1958) along with the first determined protein structure of sperm whale myoglobin by X-ray crystallography (Kendrew et al. 1958), in the same year. However, theoretical physics in long-wait could not characterize such an intrinsic property of proteins, microscopically, despite accumulated X-ray-solved structures in the 60’s and early 70’s, until computational facilities were mature enough to accomplish the first Molecular Dynamics (MD) simulation by Karplus and coworkers (McCammon et al. 1977). This seminal work described atomic motions following Newton’s 2nd law with an empirical potential energy function and suggested a fluid-like nature in the interior of the protein (McCammon et al. 1977).

Not before long, in early 80’s, Noguti T and Gō first examined the fluctuations of globular protein by a set of collective variables (Noguti T and Gō N, 1982). The new application of Normal Mode Analysis (NMA; Goldstein, 1950) to the protein BPTI deciphered the mode compositions of protein fluctuations (motions in the range <30 cm^−1^ dominate the fluctuations) and described crystallographic temperature (B−) factors (the degree of uncertainty in atomic positions) surprisingly well (Gō et al. 1983; Brooks and Karplus, 1983). Domain motions at the active sites of lysozyme and ribonuclease were seen to occur in low frequency normal modes (Levitt et al. 1985). Within the small fluctuations at the equilibrium (reached after energy-minimizing the crystal structure according to a given potential energy function), NMA approximates the complicated potential (comprised of multiple contributions including bond stretching, angle bending, dihedral, electrostatics and van der Waals) surface harmonically. The second derivatives of the potential (with respect to atom displacements), the Hessian, is singular-value decomposed to obtain the normal mode shapes (eigenvectors) and frequencies (the square root of the eigenvalues). The analytical approach, solving the eigen-problem of the 3*N_a_* by 3*N_a_* Hessian matrix (*N_a_* is the number of atoms in the protein) greatly reduces the computation time for obtaining the equilibrium dynamics of protein, as compared to MD. The low-frequency (slow) modes containing a certain degree of anharmonicity (Gō et al. 1983) not only are able to describe functional, configurational changes (Brooks and Karplus, 1985) but also help in the refinement of X-ray structures (Kidera and Gō 1992a, b).

NMA usually yields robust results, especially in the low frequency regime, because the results are not subject to statistical errors or sampling inaccuracies (unlike those retrieved from MD). However, MD, which makes no assumption about the underlying potential surfaces and allows transitions across energy barriers (which anharmonic motions include), is dearly needed to describe non-equilibrium dynamics involved in biologically important large conformational transitions, given a sufficient duration of simulations (Kitao et al. 1998; Arkhipov 2006a, b). However, the heavy computation of MD has limited its applicability to large biomolecular systems. The renaissance and further developments of coarse-grained MD (CG-MD) models, able to overcome the computational limit at a decreased resolution while maintaining key dynamic features of described systems (Tozzini, 2005), made possible simulations up to tens of microseconds (see our review on CG-MD in this series).

NMA gained unprecedented popularity in the late 90’s along with two simplified schemes that resulted in a huge reduction of computational cost. One is the introduction of the Elastic Network (EN) concept, using a much *simplified potential*, being introduced by Tirion (Tirion, 1996) who proposed modeling molecules with their atoms within an interaction range being connected by Hookean springs of a universal strength. However, the first use of the word ‘network’, interpreting protein as junctions and elastic connections, was pioneered by Bahar and coworkers (Bahar et al. 1997) who took the idea from polymer science (Flory, 1976), using only C_α_ atoms to represent the protein. The description of proteins in reduced presentations is the so-called *Coarse-Grained* (CG) approach. Slow modes derived from both schemes were found to agree well with slow modes obtained from the standard NMA that uses a much more detailed potential. The saving of computational cost is tremendous: GNM, Bahar’s model, required the diagonalization of a dimension-reduced Hessian, Γ (see below), which took 8.2 sec for T4-lysozyme (164 residues) on a single workstation (Bahar et al. 1997), as compared to 3 days by NMA (see Table 1) and much longer for nanosecond MD simulation for proteins of the same size to capture similar structural deformations.

Since then, the ease of programming and reduced computational cost due to the use of simplified potentials and smaller number of degrees of freedom resulted in wide-spread application of CG-EN models to deduce both the conformational dynamics of large structures and assemblies, including hammerhead ribozyme (Van Wynsberghe and Cui, 2005), CDK2/cyclin A (Dror and Bahar 2005), citrate synthase (Hinsen, 1999), hemoglobin (Xu et al. 2003), HIV reverse transcriptase (Bahar et al. 1999; Hinsen, 1999), hemagglutinin A (Doruker et al. 2002), aspartate transcarbamylase (Hinsen, 1999), F1-ATPase (Cui et al. 2004), an actin segment (Ming et al. 2003), GroEL-GroES (Keskin et al. 2002), the ribosome (Wang et al. 2004; Yang et al. 2006; Cui and Bahar, 2006) and viral capsids (Rader et al. 2005). Many intriguing biological systems as such, in a variety of sizes and extended applications (Tama et al. 2004; Leo-Macias et al. 2005) of ENMs have been carefully reviewed (Case, 1994; Kitao and Gō, 1999; Ma, 2005; Rader and Bahar, 2005; Tozzini, 2005). However, in-depth comparisons of the theories that underline the ENMs and their offshoots have been lacking.

In this review, ENMs (highlighted on Tirion’s model, GNM, ANM and RTB/BNM) are illustrated in sufficient theoretical details: the basic hypotheses, the physical grounds, mathematical treatments and consequently achieved computational efficiency. The well comprehended ENM foundations serve to interpret data obtained from comparisons between predictions and experimental results, namely the observed equilibrium and non-equilibrium dynamics and those within ENMs themselves. Slow normal modes derived from different potentials and molecular resolutions are found robust within a subspace spanned by 5–6 dimensions (Nicolay and Sanejouand, 2006) but not on a one-to-one basis between the models.

NMA-based models show different levels of accuracy (Tama and Sanejouand, 2002) when examining the agreement between experimentally characterized large conformational changes and single slow-mode-driven structural deformations. These results together can be understood by motions taking place in harmonic energy wells with different curvatures approximated by different potentials and coarse-grained levels in the models. The profile of potential of mean force also helps in understanding the origin of a better predicted open→close transition than the close→open counterpart by ENMs, using the concept of conformational selections (Ma et al. 1999; Dror and Bahar, 2006). Lastly, benefiting from the statistical study on comparisons of X-ray B-factors, RMSDs of NMR ensembles and GNM, we recently reported how experimentally characterized dynamics can comprise (and be affected by) motional components in different frequencies (Yang et al. 2007) and how the findings can be of use to understand the frequency dispersions of the models themselves. A separate review article on coarse-grained molecular dynamics simulations is presented back-to-back so as to address the non-equilibrium structural transitions that are beyond the reach of herein introduced EN models, given their basic hypotheses.

## Theory—The ENM Models

### Atomic-ENM

#### Tirion’s model

**Figure 1:**
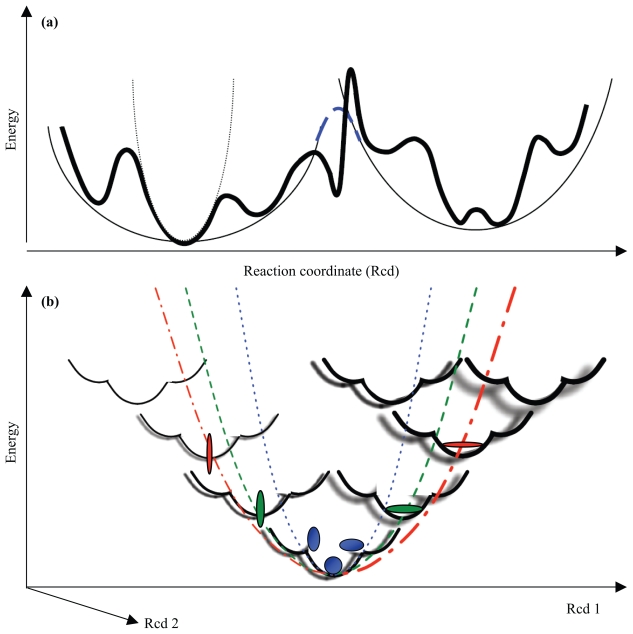
(**a**) **The effect of simplified potentials**. The energy landscape outlined by the conventional force field (detailed potential, as used by standard NMA) is drawn in thick lines. Simplified potential, (in thin lines) as used in Tirion’s or CG-EN models, approximates the rugged potential surface crossing the local energy barriers. At coarse-grained level, the rugged potential can be described by RTB/BNM and the smoothed-out one by XNM {X = A, βG, C, D and HE …}. Despite the difference between the two potentials, equilibrium dynamics characterized by X-ray and NMR can be well described by both potentials from which the derived slowest modes cover the slowest ends of experimentally observed dynamics (see Discussion). In contrast, the slowest modes derived from the smoothed-out potential are *slower* than those derived from the detailed potential due to the narrower energy wells in the latter. As a result, large conformational transitions with high anharmonicity could be better predicted by the slowest modes derived from the simple elastic potential than by force-field-based potential. The blue long dashed line joins the equal energy points of two CG energy wells as described by PNM (see Supplementary Material). (**b**) **Similarity of the** ***shape*** **of hierarchical global potential envelopes.** The thick lines indicate the actual detailed potential. The blue dotted line approximates the local energy well as in the standard NMA or ENMs. Green dashed and red dot-dashed lines approximate the potential envelopes at a higher hierarchy. The fractal-like similarity between the curvature of the local well and those of the potential envelopes at a higher hierarchy could account for part of the reason why NMA-based models, assuming a minimal structural deformation and approximating the potential of mean force harmonically at the equilibrium, can often predict large conformational changes reasonably well.

To dispense with the problematic energy-minimization process prior to NMA while gaining computer efficiency, Tirion proposes a model to connect atom pairs with Hookean springs with a universal force constant *γ* (Tirion, 1996). The equilibrium structures are taken from the experimentally (X-ray or NMR) characterized structures assuming a zero energy. The resulting potential is a harmonic approximation that is much simplified than sophisticated potentials (Fig. 1a) used in NMA involving multiple bonded and nonbonded terms, which may or may not be harmonic depending on the instantaneous configurations of biomolecules in question. The total energy *E* of a molecule is

(1) $$E_{Tirion}=\sum_{i,j=1}^{N_{a}}\frac{\gamma }{2}{\left(\left|{\vec{r}}_{ij}\right|-\left|{\vec{r}}_{ij}^{0}\right|\right)}^{2}H\left(R_{c}-\left|{\vec{r}}_{ij}^{0}\right|\right)$$

where **r⃗***_ij_*^0^ is the vector connecting atoms *i* and *j* at equilibrium, defined in the PDB structures. Atoms *i* and *j* in the molecule that contains *N_a_* atoms are connected by a Hookean spring if their separation is closer than a cutoff distance, *R_c_*. *H*(x) is the Heaviside step function that is 1 when x ≥ 0 and zero otherwise. Force constant *γ* is chosen to optimally scale with NMA results or experimental measurements, such as the temperature (B−) factors of X-ray characterized structures (Tirion, 1996; Bahar et al. 1997). ENM reproduces the frequency spectrum and the eigenvectors of low-frequency modes of NMA at a 10^−3^ computational cost of NMA’s (Tirion, 1996). The improved efficiency is attributed to the absence of the initial energy-minimization step required before applying NMA and accelerated computations for the force constant matrix (second derivatives of the potential) due to the simplified energy function (Tirion, 1996). R_c_ was tested over values of 4.5, 4.9, 5.4 and 5.9 Å (including the sum of van der Waals radii, roughly 3.4 Å, for contacting atoms) and in all cases gave satisfactory results (Tirion, 1996).

### CG-ENM

#### GNM

GNM, developed by Bahar and coworkers (Bahar et al. 1997), differs from Tirion’s ENM in the following aspects. It was the first ENM that represents proteins with interacting ‘nodes’ at the amino-acid level (the CG scheme) while successfully reproducing X-ray B-factor data (Bahar et al. 1997), H/D exchange free energy costs (Bahar et al. 1998b) and ^15^N-NMR relaxation order parameters (Haliloglu and Bahar, 1999). Its potential employs the vector form of the displacement for node pair *i* and *j* under the isotropic assumption (<ΔXΔX*^T^* > = <ΔY ΔY*^T^* > = <ΔZ ΔZ*^T^* > = (1/3) <ΔR ΔR*^T^* >, *T* is transpose, see the Supplementary Material):

(2) $$\begin{array}{l} E_{GNM}=\sum_{i,j=1}^{N}\frac{\gamma }{2}\left({\vec{r}}_{ij}-{\vec{r}}_{ij}^{0}\right)\\ •\left({\vec{r}}_{ij}-{\vec{r}}_{ij}^{0}\right)H\left(R_{c}-\left|{\vec{r}}_{ij}^{0}\right|\right) \end{array}$$

(3) $$\text{or }E_{GNM}(\Delta R)=\frac{\gamma }{2}\Delta R^{T}\Gamma \Delta R\;(\text{in matrix}\;\text{form})$$

where Δ**R** is the column vector of Δ**r⃗***_i_*; *i* runs over 1 to *N* for a protein of *N* residues; *γ* is again the uniform spring constant (force constant) and *Γ* is the *N* × *N* connectivity matrix (see Supplementary Material for details).

We can easily see the difference in the potentials of Tirion’s and GNM. The inner product of vector differences, instead of the scalar difference of the *i–j* pair separations, penalizes not only the translational but also the rotational displacement, which partially accounts for its better B-factor agreement over other ENM models (Cui and Bahar, 2006).

The *nodes* in GNM are usually the C_α_ atoms of amino acid residues. *R_c_* is generally set near or above 7 Å, the range of which covers the first coordination shell (Bahar et al. 1997; Cui and Bahar, 2006; see also Discussion). From the basics of Statistical Mechanics, one can easily derive the following results (see Supplementary Material for details)

(4) $$\begin{array}{l} \langle \Delta {\vec{r}}_{i}^{2}\rangle =\frac{3k_{B}T}{\gamma }{\left(\Gamma^{-1}\right)}_{ii}\\ \langle \Delta {\vec{r}}_{i}\cdot \Delta {\vec{r}}_{j}\rangle =\frac{3k_{B}T}{\gamma }{\left(\Gamma^{-1}\right)}_{ij} \end{array}$$

where <Δ**r⃗***_i_*^2^> is the ensemble average of the squared displacement of node *i* from equilibrium. Clearly, in GNM, only the magnitude square (the variance from the mean) of fluctuation is obtained due to the isotropic assumption and therefore the directions of the motions are not predicted. One should note that Γ has a rank of *N*−1. The diagonalization of matrix results in one zero eigenvalue and the associated trivial mode accounts for the rigid-body translation of the entire molecule. Therefore the Γ^−1^ is a pseudo-inversion that is the sum of all the non trivial-modes. The covariance for pair *i–j* can be rewritten as

(5) $$\begin{array}{l} \langle \Delta {\vec{r}}_{i}\cdot \Delta {\vec{r}}_{j}\rangle =\frac{3k_{B}T}{\gamma }{\left(\Gamma^{-1}\right)}_{ij}\\ =\frac{3k_{B}T}{\gamma }\sum_{k}{\left[\lambda_{k}^{-1}u_{k}u_{k}^{T}\right]}_{ij} \end{array}$$

*λ_k_*^−1^ is the reciprocal of the *k*^th^ nonzero eigenvalue (the frequency square of the *k*^th^ mode) solved for Γ. The slowest mode (the 1st mode, with the lowest frequency) that has the most dominant contribution to the entire fluctuation is along the eigenvector **u***_k_* that is led by the biggest *λ_k_*^−1^. The slowest modes describe functional motions that are to great biological interests (Bahar et al. 1998a; Yang et al. 2005a). In addition, it is known from crystallography that the isotropic B-factors are proportional to the sizes of the fluctuations. That is

(6) $${\text{B}}_{\text{i}}=(8\pi^{2}/3)<{\left(\Delta {\vec{r}}_{\text{i}}\right)}^{2}>$$

Hence, B-factors can be predicted from GNM whereas the needed spring constant is obtained as scaling the predictions to match up with the experiment, namely the magnitude of B-factors, assuming internal atomic fluctuations fully account for structural uncertainties (Bahar et al. 1997; Cui and Bahar, 2006). The correlation between theories and experiments on the B-factors is found around 0.6 for a wide range of cutoffs and temperatures (Yang et al. 2006, supplementary material) and is 0.65 if crystal contacts are considered (Kundu et al. 2002).

CNM, an isotropic model extended from GNM, has reported a 0.74 correlation with B-factor profiles (Kondrashov et al. 2006) of 98 high resolution (<1.0 Å) structures while employing a few modification schemes including crystal contacts (also reported previously by Kundu et al. 2002), residue contacts determined in atomic level while maintaining a *N* × *N* connectivity matrix (Γ) and enhanced force constant for backbone connections by a factor 10 (Kondrashov et al. 2006). More details can be seen in the Supplementary material.

#### ANM/Hinsen’s CG-ENM

The ‘restoration’ of predicted fluctuations from 1-D (magnitude only) to 3-D came no later than 1998, pioneered by Hinsen (Hinsen, 1998). Hinsen’s ENM (HENM) is carried out at the residual level, the potential adopts the same form as Tirion’s except for the spring constant being in exponential decay with increasing residual pair separations. The decay corresponds to a weakened interaction between pairs far apart which simply reflects the physicochemical reality, although there is no specific reason why an exponential form has to be taken (Hinsen, 1998). The suggested form is

(7) $$\gamma \left({\vec{r}}_{ij}^{0}\right)=c\times \text{exp}\left(-\frac{{\left|{\vec{r}}_{ij}^{0}\right|}^{2}}{r_{0}^{2}}\right)$$

The parameter *r*_0_ is set at 3–7 Å so as to best reproduce the low frequency normal modes obtained with the AMBER force field (Hinsen, 1998; Hinsen et al. 1999); *c* is a scaling factor. The design eliminates the need for assigning a cutoff distance (or interchangeably in this article, cutoff) as in other ENM models. However, an updated version of γ(r⃗*_ij_*^0^) takes a stronger interaction for residue pairs in separation less than 4 Å, the range of which covers well the backbone neighbors. The spring constant for **r⃗***_ij_*^0^ above 4 Å now decays with 1/*r*^6^. This format is proved to better approximate the long-time dynamics of proteins (Cui and Bahar, 2006).

Following a different path of derivation, Atilgan obtained the same result that differs from Hinsen’s only at the spring constant being set as constant for simplicity hence the need to assign a cutoff distance of interactions (Atilgan et al. 2001). ANM is basically the CG version of Tirion’s ENM except in assuming uniform mass for each amino acid (or bead) as done in HENM. HENM and ANM basically solve an eigen-problem involving a 3*N* ×3*N* force constant matrix (the second derivatives of the potential, see below), the Hessian (**H**) that contains *N* × *N* super elements **H**_ij_ (each super element is of dimension 3 ×3)

(8) $$\begin{array}{l} H_{ij}\left(\left|{\vec{r}}_{ij}^{0}\right|\right)=\frac{-\gamma }{{\left|{\vec{r}}_{ij}^{0}\right|}^{2}}\left[\begin{array}{ccc} x_{ij}x_{ij} & x_{ij}y_{ij} & x_{ij}z_{ij}\\ y_{ij}x_{ij} & y_{ij}y_{ij} & y_{ij}z_{ij}\\ z_{ij}x_{ij} & z_{ij}y_{ij} & z_{ij}z_{ij} \end{array}\right]\\ H\left(R_{c}-\left|{\vec{r}}_{ij}^{0}\right|\right)\;\;\;\text{for }i\neq j \end{array}$$

(9) $$H_{ii}=-\sum_{j=1}^{N}H_{ij|i\neq j}$$

The form is derived from the second derivatives (with respect to the node displacements) of the potential. Here, *x_ij_*, *y_ij_* and *z_ij_* are the components of **r⃗***_ij_*^0^. Six zero eigenvalues and associated eigenvectors obtained from diagonalization of the Hessian stand for the six degrees of freedom of rigid-body translation/rotation. The 3*N*-6 non-trivial eigenvectors that give sizes and directions of motions for nodes in each mode are obtained.

As for the efficiency, Hinsen’s Hessian is less sparse than ANM’s and therefore takes minimal advantage of the regular sparse matrix solver hence a slower computation than ANM, despite similar low-frequency modes being obtained in both (Cui and Bahar, 2006). **DNM**, a modified version of ANM, which uses distance-dependent force constants (hence the name Distance-based Network Model; Kondrashov et al. 2007b), was reported to have an improved prediction on Anisotropic Displacement Parameters (ADPs) over ANM (Kondrashov et al. 2007). More details are available in the Supplementary Material.

#### RTB/BNM

**Figure 2:**
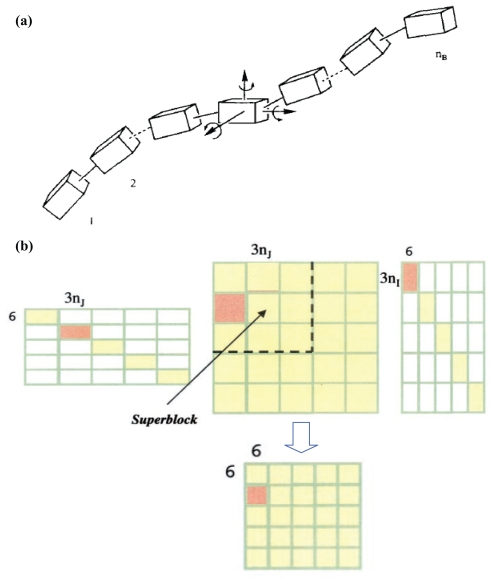
(**a**) Each block in the molecule is a rigid body that is subject to local translations/rotations (T/R) described by 6 T/R eigenvectors. The figure is reproduced from Durand et al. (1994) (**b**) The atomic Hessian matrix is expressed in a reduced basis for each coupled or diagonal block. Block *i* and *j* has *N*_a,i_ and *_N_*_a,j_ atoms, respectively. **Ui** (part of the **P** matrix) is a *N*_a,i_ by 6 matrix that consists of 6 T/R vectors, representing the rigid body motions of block *i*. The atomic Hessian elements for blocks *i* and *j* is projected to a 6 × 6 reduced Hessian **Hij^b^** using the equation **Hij^b^** = **Ui***^T^* **Hij** **Uj**. *Superblock*, used in BNM, comprises several blocks. The Hessian elements within each *superblock* is computed on the fly and then projected to reduced dimension with **P**. The figure is reproduced from Durand et al. (1994) and Li and Cui, (2002).

The collective motions seen in low-frequency normal modes often occur at the levels of residues, secondary structures, or even domains. It provides the physical motivation to describe such motions as rigid-body translations/rotations of blocks (RTB) of atoms (Fig. 2a), the mathematical treatment of which is the projection of the 3*N*_a_ by 3*N*_a_ atomistic Hessian into a small 6n_B_ × 6n_B_ *block*-matrix, where *N*_a_ is the number of atoms and n_B_ is the number of *blocks* chosen for the molecule in question (see below).

Although Bahar *physically* coarse-grained proteins, Sanejou and and co-workers were among the first to coarse-grain the protein at the *mathematical* level as early as 1994 by breaking up the protein into residue blocks (the building-block approach) while introducing rotation-translation basis into the atomic Hessian (Durand et al. 1994). With the eigen-problem solved at a reduced dimension, RTB makes the dynamic analyses of supramolecules computationally tractable, in the same spirit as other CG models. The analysis on a series of proteins of various sizes is made possible and demonstrates a good reproducibility of standard NMA results especially in the low-frequency spectrum. (Tama et al. 2000).

Atomistic Hessian herein, **H**, of size 3*N*_a_ × 3*N*_a_, is first computed and stored. The projection matrix, **P**, of size 3*N*_a_ × 6n_B_, comprising six local translation/rotation vectors of blocks (and the degrees of freedom of each block sum up to *N*_a_, see Fig. 2b), is prepared for the subsequent projection (the detailed formula for **P** can be found in Li and Cui, 2002). A block, although can be a cluster of any number of atoms, is often chosen to consist of atoms of a single or several consecutive residues in sequence (Tama et al. 2000). The projected Hessian,

(10) $$H_{b}=P^{T}\mathrm{HP}$$

of size 6n_B_ × 6n_B_, usually 25 fold smaller in memory storage and therefore 125 fold faster in computation than **H** (consider1block= 1 residue ≈ 10 atoms and in one dimension, 3*N*_a_/6n_B_ ≈ 5), is diagonalized to give 6n_B_ eigenvalues and eigenvectors (Fig. 2b). The corresponding 3*N*_a_ atomic displacements can then be approximated by projecting the solutions from a reduced dimension back to the full dimension as

(11) $$A_{p}=PA_{b}$$

Here, **Ap** is the approximated eigenvector matrix (3*N*_a_ × 6n_B_) of **H**, which consists of 6n_B_ slowest normal modes and can be projected from **Ab** (6n_B_ × 6n_B_), the eigenvector matrix of **Hb,** with multiplying the projection matrix **P**.

The Block Normal Mode (BNM) approach is basically the same as RTB, yet employs a better computational implementation such that the required atomic Hessian elements for constructing the ‘blocks’ are computed on the fly and the big **H** never has to be stored (Li and Cui, 2002).

Note that approaches such as RTB/BNM are different from other CG-EN models on two aspects. RTB and BNM inevitably need the preliminary energy minimization, as the standard NMA, before building the atomic Hessian elements. Moreover, the harmonic potential they describe, despite being *blocked*, is less smoothened out than models such as GNM or ANM that have a physically coarse-grained elastic potential (Fig. 1a, see also Discussion).

Other EN Models such as backbone-enhanced elastic network model (BENM), β Gaussian Model (βGM), quantized elastic deformation model (QEDM), plastic network model (PNM), double-well elastic network model (DWNM) and models based on linear response theory, also to readers’ great interest, are introduced in the supplementary material. Their potentials and resulting properties of Hessians are summarized in Table 1.

#### Online access of the CG-EN models and NMA results

Web services for GNM (Yang et al. 2005b, 2006), ANM (Eyal et al. 2006), NMA (Wako et al. 2004) and others (see a review by Xiong and Karimi 2007) are developed in recent years to facilitate a high-throughput analysis on conformational dynamics via ‘biologist-friendly’ interfaces.

## Discussion

The section is composed to first analyze the basic assumptions of EN models, the nature of the normal modes that are derived from them and the nature of the experimental observations that are often compared with ENM-derived predictions and eventually answer the question—which EN model is the ‘best’?

### The essence of the cutoffs

The cutoff distance (*R_c_*) is usually set to take account the physical reality and save the computation cost on those negligible interactions for atom pairs far apart (Leach, 2001). For Tirion’s ENM or ANM, *R_c_* was first chosen to best reproduce the frequency spectrums of NMA or GNM respectively (Tirion, 1996; Atilgan et al. 2001) whereas in GNM, it was chosen to include the contacts within the first coordination shell defined by the C_α_-based radial distribution function (Bahar et al. 1997). However, Yang has shown that a range of *R_c_* from 7 to 15 Å simply renders statistically identical correlations with B-factors over 1250 nonhomologous proteins (Yang et al. 2006). The robust isotropic nature of time-average fluctuations was also reported in Eyal and Kondrashov’s studies (Eyal et al. 2007; Kondrashov et al. 2007).

Taking the correlation with B-factors of Protein/DNA/RNA biocomplexes as a function of residue-residue contact distance (*R_c_*), nucleotide-nucleotide contact distance (*R_p_*), residue-nucleotide contact distance ((*R_c_* + *R_p_*)/2) and the number of beads (from 1 to 3) used to represent a nucleotide given one bead per protein residue, Bahar and coworkers found that the result was maximized at *R_c_* = *R_p_* = 7 Å given 3 beads per nucleotide which is known to be roughly 3 times heavier than an amino acid (Yang et al. 2006). The setting of 3-nodes-per-nucleotide (P, C4* in the sugar and C2 in the base) plus 1-node-per-residue also made nodes distributed more evenly within the shape of the molecule than other settings.

The use of cutoff distance in these models simply serves to measure the local packing density (Halle, 2002) which is the counts within a fixed volume (consequently a fixed cutoff distance). The concept herein has been widely used in classical/statistical mechanics for sampling particle properties at a coarse-grained level (Nitzan, 2006). Mixed cutoff schemes, as first attempted in the study above, do not sample such a density in *equal volume* while the packing density is known to have a dominant contribution to residue fluctuations (Halle, 2002; Cui and Bahar, 2006; Yang et al. 2006). Biased local density sampled leads to an unphysical Hessian that preserves no cutoff information, causing impaired predictions. Hence, as long as the employed cutoff renders *a good representation of local features* (not too small for nodes to ‘see’ only the backbone neighbors or too wide for all the nodes to be connected together), similar prediction results for isotropic data are faithfully obtained. We should also note that anisotropic vibrations are more sensitive to employed cutoffs hence models based on detailed potentials giving better predictions for ADPs than ANM does (Kondrashov et al. 2007).

### Slow modes rather than fast modes are robust

Nicolay and Sanejouand asked how many normal modes are needed for a given NMA-based model to describe the normal modes obtained from other protein models that use different potential and coarse-grained schemes. The results suggested that 5–6 Tirion’s EN modes in the lowest frequencies are enough for the description of a few slow modes obtained with the all-atom CHARMM potential (Nicolay and Sanejouand, 2006). The invariant nature of a robust subspace spanned by 5 to 6 normal modes was again seen in the crosscheck over the other two CG-EN models, including ANM (Nicolay and Sanejouand, 2006). Moreover, low-frequency subspace from essential dynamics analysis is found to be spanned well by a few low-frequency normal modes (Rueda et al. 2007). In fact, similar slowest (1st) modes can be obtained through a hierarchy of coarse-grained (HCA) schemes for a given EN model (Doruker et al. 2002; Ming et al. 2002).

Proteins with a similar architecture encode similar conformational dynamics, as natural as one might expect. However, slow components are more robust against structural variations than the fast ones (Keskin et al. 2000; Cox et al. 2007). Quantitatively speaking, a 2.1 Å RMSD between two structures of the same protein, separately solved by X-ray and NMR, gives a correlation of 0.94 (statistical average) between their slowest-mode profiles that are derived from GNM (Yang et al. 2007). The insensitivity to minor structural changes is understood to stem from the collective nature of the low-frequency modes. The collective oscillation is a joint effect of many interacting pairs, summed up to approach a universal form that is governed by the central limit theorem, regardless of the details of pair positions or potentials (Tirion, 1996; Atilgan et al. 2001). Another interesting observation made by ANM combined with a structural perturbation method is that low modes are robust to sequence variations or in other words, insensitive to mutations (Zheng et al. 2006).

### Magnitude rather than directions of fluctuations is a robust feature

On the other hand, the fluctuation *magnitude* is better predicted than the *direction* of the motions. Kondrashov used five different CG-EN models including BNM using the CHARMM potential to examine their agreement with crystallographically characterized isotropic and anisotropic dynamics (Kondrashov et al. 2007). The result showed almost the same correlation between the predicted time average magnitude and the reported isotropic fluctuations for the five models, whereas the predictions on the reported directions of motions are shown to be model-dependent (Kondrashov et al. 2007). Bahar and coworkers confirmed the same observation in a systematic study on a collection of ADPs (see the DNM model in Theory) reported in 93 high-resolution PDB structures and found the sums of the diagonal elements (the magnitude) in the inverse Hessian to agree better with experiment than the off-diagonal elements (indicating the directions) (Eyal et al. 2007). In fact, Kondrashov and Eyal found experimentally reported ADPs are highly refinement package dependent (average anisotropy given by Refmac is 0.64 and is 0.51 by SHELX; Kondrashov et al. 2007) and greatly sensitive to the forms of crystal packing symmetry (substantial difference in ADPs reported for the same proteins packed in different space groups; Eyal et al. 2007). One should note that a model that is tuned to best predict the directions of motions does not necessarily best describe the magnitude of the motions (Kondrashov et al. 2007; Eyal et al. 2007), indicating strong experimental artifacts. Use of ANM to predict RMSDs of the 64 NMR ensembles also found better agreements in the magnitude (0.69) rather than the directions (0.62) (Yang et al. 2007). The better reproducibility in the magnitude rather than the directions is not only seen between experiment and theory but also between theoretical results (Cox et al. 2007).

### Understanding dynamics hidden in the electron cloud

In X-ray crystallography, the iso- and anisotropic B-factors are obtained via a fitting process to position the atoms that best represent the electron density distribution. They have been understood more as the structural uncertainty (or errors) rather than quantization of dynamics. The difficulty to fully count B-factors as dynamic quantities is that they contain strong contributions from the crystal packing. In the early 90’s, Kidera and Gō have shown through the use of the standard NMA that the external contribution (58%) to the B-factors are actually larger than the internal ones (42%) in human lysozyme (Kidera and Gō 1992 a, b). As EN models describe internal fluctuations, only, how can a model like GNM score a good correlation with B-factors?

The reason is explained as follows. We have to note that GNM is a 1-D model that motions are carried out in the 1-D magnitude space with a rigid-body translational shift (the trivial mode led by a zero eigenvalue) for the entire molecule. B-factors can therefore be fitted as

(12) $$B_{i}^{iso}=c_{trans}+c_{NM}\sum_{k=2}^{N}{\left[\lambda_{k}^{-1}u_{k}u_{k}^{T}\right]}_{ii}$$

As a result, the correlation between the *profiles* (as a function of residue index) of *B_i_^iso^* and $\sum_{k=2}^{N}\left[\lambda_{k}^{-1}u_{k}u_{k}^{T}\right]$ will not be changed no matter how big or small the constant *c_trans_* is. Parameter *c_NM_* is a constant that contains *γ*. On the other hand, for 3-D models like ANM, contributions from rotational rigid body motions should be considered.

(13) $$\begin{array}{l} B_{i}^{iso}=c_{trans}+c_{rotate}\times {\left\|{\vec{r}}_{i}-{\vec{r}}_{mc}\right\|}^{2}\\ +c_{NM}\sum_{k=2}^{N}{\left[\lambda_{k}^{-1}u_{k}u_{k}^{T}\right]}_{ii} \end{array}$$

Where **r⃗***_i_* and **r⃗***_mc_* are the position vectors of atom *i* and mass centroid of the molecule respectively. This is the minimal fitting scheme using the least parameters. Of course, due to the heterogeneity in the crystal, popular models (Winn MD et al. 2001) using more parameters is quite understandable. Also, if considering how each mode could be excited by different crystal packing forms, a modified version of the above equation would be to parameterize the contribution of each normal mode (Song and Jernigan, 2007). Both the rigid-body rotation (||**r⃗*i*** − **r⃗*mc***||^2^) and internal vibrations $\left(\sum_{k=2}^{N}{\left[\lambda_{k}^{-1}u_{k}u_{k}^{T}\right]}_{ii}\right)$ contribute to the shape of the theoretical profiles. However, most of the comparisons between ENM-derived internal fluctuations and B-factors are done without considering such a contribution of rigid-body rotation (Kondrashov et al. 2007; Eyal et al. 2007). This could be part of the reason that 3-D ENM models compare slightly worse with B-factors than 1-D models do (such as GNM and CNM) on top of the acknowledged fact that GNM penalizes the rotational deformation when 3-D ENMs do not (see the GNM subsection in Theory; Bahar, 1997; Cui and Bahar, 2006).

### Understanding NMR characterized dynamics

NMR characterizes protein structure and dynamics in the solvated state. Predictions from ENMs have been in a good agreement with such NMR-characterized dynamics, namely the order parameters (Yang and Kay, 1996), derived from NMR relaxation data (Haliloglu and Bahar, 1999; Ming and Bruschweiler, 2006). Accordingly, Chen recently uses such quantity as a benchmark to rationally select ensembles from MD snapshots that best reproduce the order parameters (Chen et al. 2007). In addition, it is interesting to see that GNM has a 0.74 correlation with the RMSDs of NMR ensembles as opposed to a 0.59 correlation with the B-factors of their X-ray counterparts (same proteins alternatively solved by X-ray). Deleting the slowest GNM mode that contributes to the time-average fluctuations and then comparing with the same aforementioned quantities leaves the correlation with X-ray unchanged but dramatically decreases the correlation with NMR, indicating the differences in the spectrum of modes accessible in solution and in the crystal environment. Specifically, large amplitude motions sampled in solution are subdued in the crystalline environment of X-ray crystallography due to the restraints from crystal contacts (Kundu et al. 2002) and low temperatures (Yang et al. 2007).

Refined NMR conformers are obtained from simulated annealing runs and energy minimization (Brünger, 1991a, b) over the detailed potential surface defined by the target function (Schwieters et al. 2003) that comprise both the empirical force field and NMR restraint-derived penalty terms (Yang et al. 2007). Although more studies are needed for a clear understanding of the correlation between NMR and GNM, surprisingly, anharmonic procedures as such to populate the NMR conformers in distributed local wells can be approximated by GNM that uses simplified elastic potential. The statistical result suggests NMR ensembles should not be deemed solely as the range of ‘errors’ in structure determination but more as a set of conformations accessible to the molecule in question under the experimental conditions.

### Open-to-close transitions being better predicted than contrariwise

There have been studies showing protein open→close transitions (meaning that the open form of the structure is used by NMA- or MD-based models to generate low frequency normal or PCA modes in order to compare with experimentally identified structural transition vectors) are better predicted than their close→open counterparts in quite a few systems including adenylate kinase (Temiz et al. 2003; Miyashita et al. 2003; Maragakis and Karplus, 2005), citrate synthase (Hinsen, 1999), LAO binding protein (Tama and Sanejouand, 2001), hemoglobin T→R2 transition (Xu et al. 2003) and E. coli ABC Leu/Ile/Val transport system (Trakhanov et al. 2005). A systematic study over 10 structure pairs (open/close) further confirmed this intriguing tendency (Tama and Sanejouand, 2001). The statistics in average, when ANM is used, is 0.58 and 0.43 for open→close and close→open, respectively (Tama and Sanejouand, 2001). The trend does not seem altered when different models or potentials are used. For adenylate kinase, which undergoes large, functional conformational transition that is crucial for life-related signaling cascades when triggered by hormone or metabolite cues, the correlations between prediction and experiment for open→close transition when simplified or detailed potentials are used in the ENM are 0.62 and 0.53 respectively, while those for close→open are 0.38 and 0.37, respectively (Tama and Sanejouand, 2001).

**Figure 3:**
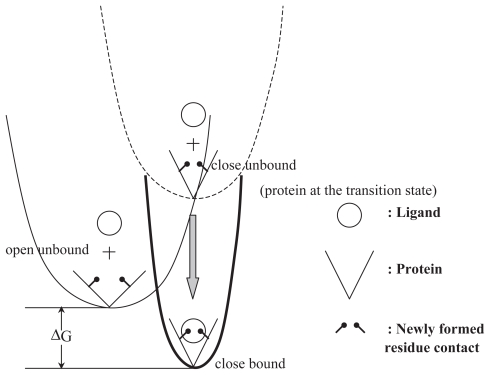
Conformational selection (Ma et al. 1999; Dror and Bahar, 2006) explains why open → close is easier predicted than close → open. Assuming only the protein takes the conformational change but ligand does not in either the bound or unbound state, the binary system, ligand + protein, evolves along the energy landscapes defined by (1) protein conformational change (with or without the contact of ligand) and (2) the binding energy ΔG, only. The conformational change is approximated harmonically by either atomic- or CG-ENM. “Close bound” state herein is referred to as ‘close state’ in the literature. Protein at the ‘open’ state access *a close but unbound* state (Dror and Bahar, 2006) along the smoothest deformational path (thin line), namely the slowest few normal modes. The protein in the disfavored “close unbound” state may further change the conformation a bit as being ‘induced’ by the ligand which then draws the whole binary system down to a new energy funnel at the big ΔG relief, in the end of the ligand docking. Since the architecture of protein is redefined by the newly formed contacts (Fig. 1 in Tama and Sanejouand, 2001), in either the “close unbound” or “close bound” (more so) state, the energy profiles (dash and solid lines, respectively) change their shape and curvature (mostly narrower) and the groups of atoms that undergo collective motions in the path open → close may not be identifiable again in the path close → open as NMA being performed on both of these close states. Not until the catalytic reaction on the substrate is complete or the ligand is released upon other chemical cues and in turn ‘pushes’ the structure back open, anharmonically, does the protein architecture resume its ‘open’ state again.

The reason is explained in Figure 3. The concept of *conformational selection* (Ma et al. 1999; Dror and Bahar, 2006) states that protein binds its ligands/substrates at its preexisting equilibrium state and therefore a certain conformational state is ‘selected’ or being ‘locked up’. Hence, an unbound structure has natural tendency to deform along the slowest normal modes to a state that resembles the bound conformation before the ligand comes in and locks the very state. For structure in the bound state, new contacts (or bonds) are formed not only at the binding site but also throughout the distal domains. The newly defined architecture due to altered pair contacts gives a Hessian distinct from that of the open state. The ‘open’ structure is therefore hardly found along the smoothest path (at the lowest energy cost) of the narrowed energy well (see Fig. 3) of the ‘close’ structure. The disallowed returning journey back to ‘open’ is permitted again upon the ligand release/bond breakage or the second incoming chemical cues.

### Which CG model is the best?

Since the late 90’s people regained interest in NMA-based models, due to aforementioned simplifications, the initial of ‘X’NM has had a decent coverage over the 26 alphabets. A natural question that arises is: which one is the best? To answer this, we shall first define what ‘good’ is? For a long time, ‘good’ has been acknowledged as reproducing (1) results derived from detailed, atomistic potentials (NMA or MD) and/or (2) experimental results (spectroscopic data, free energy measurements etc). Depending on the type of questions in study, (1) and (2) are not necessarily always the same thing (see below).

Great efforts have been expended on CG-models to reproduce the results obtained from detailed (force-field-based) potentials (Hinsen, 1998; Tama et al. 2000; Li and Cui, 2002). The coarse-grained schemes therein seem motivated from a purely computational point of view. Is there any *physical* insight gained from coarse-graining and its concomitant simplified potential used, besides the mathematical convenience?

### Simplified and detailed potentials

Although slow normal modes derived from different potentials and molecular resolutions are found robust within a subspace spanned by 5–6 dimensions (Nicolay and Sanejouand, 2006), the correspondence between modes of different models is not on a one-to-one basis. Kondrashov examined five CG models and found that BNM/RTB (using detailed potential) gave lower mode-to-mode correspondences with the other three CG-EN models investigated in the study than those within CG-ENM themselves: 15–25% lower in the *lowest* 17 modes (Kondrashov et al. 2007: Fig. 3, statistical results from 83 proteins). In fact, ANM and BNM showed the largest differences in the study: ‘only’ 0.6 to 0.7 agreements were seen between them in the slowest three modes (Kondrashov et al. 2007). Tama and Sanejouand also demonstrated that the simple potential used in ANM actually outperformed the detailed one used in RTB in predicting the protein open→close conformational transitions for 4 out of 5 proteins (Tama and Sanejouand, 2001).

On the other hand, ANM and BNM show identical accuracy in reproducing isotropic displacements (B-factors) although BNM outperforms ANM in predicting ADPs (Kondrashov et al. 2007). Note that to predict B-factors or ADPs requires a summation of all the normal modes. A question that follows is why the sum of all the modes of ANM and BNM agree with B-factors equally well when they differ in their slow modes that should contribute the most to overall fluctuations?

Recently, Bahar and coworkers demonstrated that deleting the slowest mode of GNM does not deteriorate its theoretical agreement with crystallographic B-factors due to the slowest motions being restrained by crystal contacts at low temperature (Yang et al. 2007). A subsequent study along this line, for the same set of 64 proteins, has shown that consecutive deletions of the slowest 8th modes in ANM and > 140th modes in Tirion’s model are needed before a reduced agreement with B-factors can be seen (unpublished data). This indicates that the lowest frequency components are not required for a good prediction of isotropic motions of molecules *in the crystal* although adding those components back barely (if any) decrease the correlations. This more or less explains why almost all the models give reasonable predictions on B-factors.

### Coarse-grained and fine-grained ENMs

GNM, ANM and Tirion’s ENM have different curvatures in their ‘slowest’ harmonic wells, the curvatures of which are simply captured by the second derivatives of the potentials in the Hessian(s) spanned by the slowest mode(s). However, they show nearly identical accuracy to reproduce B-factors (Eyal et al. 2007). The reason of that can be understood similarly as for simplified and detailed potentials. The study in influenza virus hemagglutinin A (Doruker et al. 2002) nonetheless shows that reduced representations of molecules produce similar shape of slow mode profiles. In fact, the slower the modes are, the more similar they are with each other across a hierarchical, reduced representation. Other evidence shows that the slowest 50 modes derived from fine-grained model (Tirion’s) or from coarse-grained model (ANM) can drive the docking of high-resolution structures into the corresponding low-resolution electron-density maps (Tama et al. 2004; Delarue et al. 2004; Hinsen et al. 2005) equally well in the Normal Mode Refinement (Kidera and Gō, 1992a, b).

### Harmonic approximations of potentials used in ENMs

The observed difference between detailed-potential- and simplified-potential-derived normal modes is a natural result of harmonic approximations taken at energy minima with different curvatures. The difference remains even when the atomistic Hessian being projected into reduced subspace in the RTB/BNM.

So, which model is the best? For structures staying *near their equilibrium states* where the dynamics can be characterized by NMR or X-ray, almost all the models perform equally well. GNM and Tirion’s ENM predict the size of RMSDs of NMR ensembles equally well (0.74) and slightly outperform ANM (0.68) (Yang et al. 2007) whereas GNM, ANM, Tirion’s ENM, βGM and standard NMA predict isotropic B-factors (or the trace magnitude of the anisotropic fluctuations) equally well with a correlation from 0.55 to 0.59 when the crystal contacts are *not* taken into account (Yang et al. 2006; Eyal et al. 2007).

Large conformational transitions that span across multiple local wells or a hierarchy of energy wells are beyond the reach of harmonic approximations discussed herein. Atomistic- or CG-MD are better approaches to study such transitions (usually accompanied with partial unfolding; see Okazaki et al. 2006) although CG-ENM could still give reasonable predictions along their slowest motional path due to the similarity of the *shape* of hierarchical global potential envelopes and the approximations by simplified potentials (Fig. 1b; Hinsen, 1998; Tama and Sanejouand, 2001; Tama et al. 2004; Cui and Bahar, 2006), which exhibits a fractal character. More rigorous systematic studies are needed to examine how the difference in the slowest normal modes from different CG-EN models impacts the prediction accuracy in dynamic events at an extended time scale.

Note that slow modes obtained from Principle Component Analysis (PCA) on MD trajectories can well agree with the slow modes obtained from both standard NMA (Kidera et al. 1992b; Kitao et al. 1998) and CG-ENMs (Doruker et al., 2000; Rueda et al. 2007) as long as sufficient length of the simulation is carried out (Kitao et al. 1998). CG-MD models are subject to the sampling problems as much as seen in conventional atomistic-MD simulations but more capable of overcoming such problem given the advantage of much enhanced computational efficiency (see our back-to-back paper in this issue).

#### Limitation of CG-ENMs

As mentioned, NMA-based models, at fine or coarse-grained levels, are not as valid in handling large configurational changes in protein, which demand crossings of multiple energy barriers, as handling small changes, due to their harmonic approximations for energy minima at equilibrium. However, large conformational changes are generally predicted well along the slowest few normal modes for the aforementioned reasons (see end of the last section). On the other hand, coarse-graining inevitably has inherited problems. As in all the CG models, the dynamics that occur within the level of coarse-graining are not sampled; for instance, the bond vibrations or the side chain reorientations cannot be evaluated in residue-based CG-ENMs. The restoration from CG to full atomic details involves the reconstruction of the backbone atoms and then side chain atoms, which pays computationally. The development of methodology as such is nevertheless nicely addressed (Heath et al. 2007).

#### Closing remarks

NMA-based methods, despite the limitation stated above, describe well the equilibrium motions. As for X-ray or NMR-characterized dynamics, the Tirion’s or CG-EN models seem sufficient to cover the slowest end of such motions. The deletion of the slowest GNM mode does not hurt the correlation between predicted and experimental B-factors. In fact, the correlation continuously goes up (although moderately) in sequential deletion of the first 10 slowest modes in ANM before it decays back down (unpublished data). Use of simplified or detailed potentials do not change much (if any) of the agreement with experiment. On the other hand, the understanding of multi-barrier-crossing conformational changes that involve partial unfolding and/or induction/perturbation from ligand demands the study from more sophisticated methods such as conventional atomistic-MD, CG-MD or theories such as LRT.

## Supplementary Material

### Other EN-models

#### BENM

Ming and Wall proposed a method to rationally optimize the parameters used in a CG-EN model by minimizing the Kullback-Leibler divergence (KLD) between the coarse-grained Hessian (from ANM) and the atomistic Hessian (from NMA) (Ming and Wall, 2005). They found that the frequency spectrum was nicely reproduced by the CG-EN model if the backbone constraints can be enhanced by a factor of 42 (Ming and Wall, 2005). They termed this model a Backbone-Enhanced-Network-Model (**BENM**) (Ming and Wall, 2005). The optimized model was found to have similar cutoff and force constant as used in ANM.

#### β Gaussian Model (βGM)

βGM is granted the name for residue interactions in the model centering not only at C_α_ but also at C_β_ atoms. The matrix remains a size 3N × 3N while the potential is added from interactions between C_α_ -C_α_, C_β_ - C_β_ and C_α_ -C_β_ (Micheletti et al. 2004). An interesting point is that the position of C_β_ can be *predicted* with good accuracy given the *i* − 1, *i* and *i* + 1 positions of the C_α_ trace (Park and Levitt, 1996) so the only needed information is the positions of C_α_ trace. βGM outperforms ANM and underperforms GNM in its agreement with B-factor profiles of X-ray structures and the RMSDs of NMR ensembles for a selected set of proteins (Micheletti et al. 2004), though the difference between the models are within the statistical errors. The time-averaged fluctuations predicted by βGM have a 0.8 correlation with the results from a 14-ns MD simulation (Micheletti et al. 2004).

#### CNM

An isotropic model extended from GNM, has reported a 0.74 correlation with B-factor profiles of 98 high resolution (<1.0 Å) structures (Kondrashov et al. 2006) while employing several modification schemes. The crystal contacts are considered as in Kundu’s work (Kundu et al. 2002). In contrast to GNM, the model couples two residues if any heavy atoms from each are found within 4.0 Å apart. An enhanced force constant for backbone-connections (bb-cons) is added to reflect the chemical reality (also used in Hinsen’s ENM and BENM, see below) hence the name Chemical Network Model (Kondrashov et al. 2006). Although residue contacts are defined using atomic information and spring constants are assigned based on the connectivity of beads, the **Γ** remains an *N* × *N* dimension as used in GNM. The nearest atom cutoff and the force constant ratio of non-bb-cons to bb-cons are explored to maximize the correlation with experimental B-factor profiles. The optimal values found for (cutoff distance, non-bb-con/bb-con) are at (4.0 Å, 0.1) and (4.5 Å, 0.05), both giving the same maximal correlation 0.74 (Kondrashov et al. 2006). The 10 to 20-fold coupling enhancement for backbone neighbors is in the same magnitude as the enhancement factor, 42, reported by Ming in his BENM model (see above). The result more or less reflects the fact that a covalent bond (~350 kJ/mol) is roughly ~12 times stronger than a hydrogen bond (~30 kJ/mol), the latter being one of the most common stabilizating forces for non-local interactions in biomolecules (Jeffrey, 1997).

#### DNM

A modified version of ANM, uses distance-dependent force constants hence the name Distance-based Network Model (Kondrashov et al. 2007). As in the CNM, the separation of pair residues in DNM is defined as the contact distance of their nearest atoms. For different such distances that fall in each of *a priori* defined distance bins 0–2.3Å, 2.3–3.3Å, 3.3–5.0Å, 5.0–7.0Å, 7.0–9.0Å and 9.0–11.0Å, corresponding spring constants (γ) are assigned for interacting pairs. To regard the chemical reality that is to have the spring constants in each bin decrease with the growing separations without introducing extra parameters, spring constant γ*_a_* is set to be 1/tr(Γ*_a_*). Here, *a* denotes the bin class. Γ*_a_* is the GNM connectivity(or contact) matrix defined at the cutoff distance *R_c,a_*, the separation range used in bin *a*. Hence, the nearer the separation (accompanying with less contact neighbors), the stronger force constant (1/contacts) of spring is assigned. A systematic study comparing 5 EN models’ reproducibility of crystallographic Anisotropic Displacement Parameters (ADPs) found that DNM gives good descriptions of molecular anisotropic movements (Kondrashov et al. 2007).

#### QEDM

Quantized Elastic Deformational Model (QEDM), first proposed by Ma and coworkers, invited attention to the possibility of describing protein dynamics in the absence of amino acid sequence and atomic coordinates (Ming et al. 2002). The main point is to take rigorous account of the protein architecture, described by the inter-residue contact topology, using the EN formalism. ANM is used herein, although the approach can easily extend to GNM. Low-resolution structures with determined electron density distributions by either X-ray or Cryo-EM are first obtained. The density maps from X-ray or Cryo-EM are clustered into *quantized* nodes that best represent the *shape of such distributions* by minimizing an error function by Topology Representing Network algorithm (see also Arkhipov et al. 2006a, b; Martinetz and Schulten, 1994). A set of *N_n_* evenly distributed quantized nodes are obtained (*N_n_* could be *N*) and ANM can be applied upon those using a certain cutoff that reasonably samples the local packing density for each node.

The approach successfully reproduces the slow modes derived from high resolution structures (Ming et al. 2002). The study lends support to the view that proteins possess mechanical characteristics uniquely defined by their particular architectures, regardless of the chemical properties (Ming et al. 2002; Yang and Bahar, 2005a).

#### PNM

Plastic Network Model (Maragakis and Karplus, 2005) and Double-Well Network Model (**DWNM**; Chu and Voth, 2007) were motivated from an interest at describing conformational transitions. Instead of using a few NMA modes to deform the structures iteratively towards the targeting conformations (Miyashita et al. 2003, 2005), the pathway of conformational transitions herein is searched by numerical procedures in PNM. Structure in each conformer well possesses elastic energy as shown in Table 1. The wells are energetically connected at their common energy point and the ‘crossing’ part is made differentiable using an analogy to the quantum mechanical convention of coupling two potential energy surfaces (eq. P1; Fig. 1; see also Okazaki et al. 2006 who adopted a similar approach in creating multi-basin CG-MD models). The minimum energy path (MEP) is then searched by the steepest decent at the saddle point (the joints of the wells) towards the minima of the wells by minimizing the integration of *G* (being the combined potential over *G*_1_, *G*_2_, …, *G_m_*) along the path using CHARMM modules (Brooks et al. 1983).

$$G=\frac{G_{1}+G_{2}-\sqrt{{\left(G_{1}-G_{2}\right)}^{2}+4{ɛ}^{2}}}{2}$$

where *ɛ* is a small number

The equation gives the solution G for the simplest case of two neighboring conformers, 1 and 2, where at the hypersurface points *G*_1_ = *G*_2_.

#### CG model using Linear Response Theory (CG-LRT)

Ikeguchi had demonstrated a beautiful way to model ligand-induced conformational change using Linear Response Theory (Ikeguchi et al. 2005).

$$\Delta {\vec{r}}_{i}≅\beta \sum_{j}<\Delta {\vec{r}}_{i}•\Delta {\vec{r}}_{j}>{\vec{f}}_{j}$$

Δ**r⃗***_i_* is the ligand-induced displacement of atom *i* (C_α_ in the CG scheme). Atoms *j* are among those strongly interacting with the bound ligand(s) that applies a force **f⃗***_j_* on *j. β* is a scaling constant. The effect of binding on *j* affects *i* through the covariance <Δ**r⃗***_i_* • **r⃗***_j_* *>*, the *ij* element of a time-independent variance-covariance matrix that is obtained from either CG-ENM (the ensemble average) or Principle Component Analysis (PCA) on MD trajectories (the time average). An excellent agreement of the predicted conformational changes with the experiment for F_1_-ATPase has been reported (Ikeguchi et al. 2005).

### Assumptions and Derivations for GNM Theory in Details

The derivation of the section starts from the definition of *ensemble average* (or expectation value) of a certain physical quantity represented as a random variable; the *probability* associated with each instantaneous value of such quantity can be defined by the *potential* of the system at the value through Boltzmann relation (see S3 below). The potential featured in GNM here is a simple, residue-based, pairwise potential.

Within the classical limit, the ensemble average of a physical quantity *A* takes the form:

(S1) $$\langle A\rangle =\frac{\int AP(A)dA}{\int P(A)dA}$$

**Figure S1.**
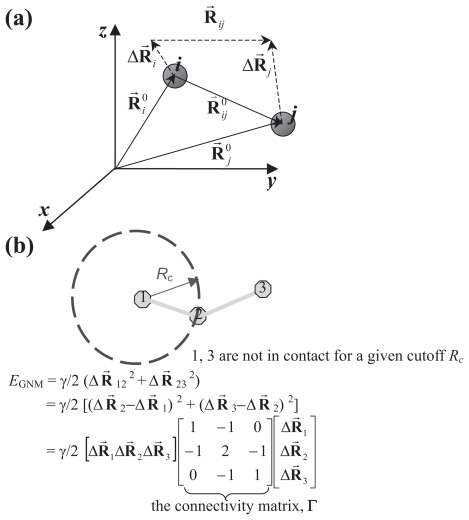
(**a**) The relative orientations of vectors **R⃗***_ij_*, **R⃗***_ij_*^0^and Δ**R⃗***_i_* (b)The elastic potential of a tripeptide molecule, represented by C_α_ atoms only. The cutoff *R_c_* here is set small enough so that only the atom pairs (1,2) and (2,3) are in contact but not for (1,3). Practical GNM cutoff often takes a value between 6.5 and 15 Å. Provided a certain pair *ij* (*i* ≠ *j*) is in contact (their linear separation <*R_c_*), the off-diagonal element *ij* of the connectivity matrix **Γ** is set to −1; otherwise 0. The diagonal elements are the negative sums of the off-diagonal elements in each individual row (or column; since **Γ** is symmetric).

Since our main concern is the fluctuation, understood as the positional variance,

let *A* be Δ**R**Δ **R*T***, which is the positional variance matrix for all the degrees of freedom (*N*) of the molecule.

(S2) $$<\Delta R\Delta R^{T}>=\frac{\int \Delta R\;\Delta R^{T}\;P(\Delta R\Delta R^{T})\text{d}\Delta R}{\int P(\Delta R\Delta R^{T})\text{d}\Delta R}$$

where $\Delta R^{T}=\left[\begin{array}{ccccc} \Delta {\vec{R}}_{1} & \Delta {\vec{R}}_{2} & \Delta {\vec{R}}_{3} & \ldots & \Delta {\vec{R}}_{N} \end{array}\right]$, Δ**R⃗***_i_* is the position vector indicating the deviation from the equilibrium state for the atom *i* (see the following and Fig. S1) and

(S3) $$P(\Delta R\Delta R^{T})\;\text{or }P(\Delta R)=\text{exp}(-E(\Delta R\Delta R^{T})/k_{B}T)$$

following the Boltzmann relation.

The energy *E* here takes the form

(S4) $$\begin{array}{l} E_{GNM}=\sum_{i,j=1}^{N}\frac{\gamma }{2}\left({\vec{R}}_{ij}-{\vec{R}}_{ij}^{0}\right)•\left({\vec{R}}_{ij}-{\vec{R}}_{ij}^{0}\right)\\ H\left(R_{c}-\left|{\vec{R}}_{ij}^{0}\right|\right) \end{array}$$

Here **R⃗***_ij_* and **R⃗***_ij_*^0^ are the positional vectors pointing from C_α_ atom *i* to C_α_ atom *j* at an instantaneous moment and at the equilibrium state (readily obtained from solved X-ray or NMR structures) respectively. The difference of vectors **R⃗***_ij_* and **R⃗***_ij_*^0^ can also be understood as the difference between vectors Δ **R⃗***_j_* and **R⃗***_i_*. A schematic illustration for the relation of these vectors can be found in Figure S1a. Rewriting the above expression into a matrix-vector form, one can easily obtain

(S5) $$E_{GNM}(\Delta R\Delta R^{T})\;\text{or }E_{GNM}(\Delta R)=\frac{\gamma }{2}\Delta R^{T}\Gamma \Delta R$$

An illustrative example of a simple tripeptide molecule to show how Γ is obtained as well as the transformation from the scalar form to the matrix form of the potential can be found in Figure S1b. Substitute (S5) into (S3), we obtain

(S6) $$\begin{array}{l} <\Delta R\Delta R^{T}>\;=\\ \frac{\int_{-\infty }^{\infty }\Delta R\;\Delta R^{T}\text{exp}(-\frac{\gamma }{2k_{B}T}\Delta R^{T}\Gamma \Delta R)\text{d}\Delta R}{\int_{-\infty }^{\infty }\text{exp}(-\frac{\gamma }{2k_{B}T}\Delta R^{T}\Gamma \Delta R)\text{d}\Delta R} \end{array}$$

With the isotropic assumption:

(S7) $$\begin{array}{l} <\Delta X\Delta X^{T}>\;=\;<\Delta Y\Delta Y^{T}>\;=\;<\Delta Z\Delta Z^{T}>\\ =(1/3)<\Delta R\Delta R^{T}> \end{array}$$

One can similarly obtain

(S8) $$\begin{array}{l} <\Delta X\Delta X^{T}>\;=\\ \frac{\int_{-\infty }^{\infty }\Delta X\;\Delta X^{T}\text{exp}(-\frac{\gamma }{2k_{B}T}\Delta X^{T}\Gamma \Delta X)\text{d}\Delta X}{\int_{-\infty }^{\infty }\text{exp}(-\frac{\gamma }{2k_{B}T}\Delta X^{T}\Gamma \Delta X)\text{d}\Delta X} \end{array}$$

One can solve the Gaussian integral in the denominator and the integral in the numerator using the equalities $\int e^{-ax^{2}}dx=\sqrt{\left(\frac{\pi }{a}\right)}$ and $\int x^{2}e^{-ax^{2}}dx=\frac{\sqrt{\pi }}{2}a^{-3/2}$, we therefore obtain

(S9) $$<\Delta X\Delta X^{T}>=\frac{k_{B}T}{\gamma }\Gamma^{-1}$$

From S6 and S8, we can also see how the elastic form of the GNM potential grants a Gaussian distribution for the residue positions in question.

From S7, <Δ**R**Δ**R*T***> = 3<Δ**X**Δ**X*T***>; that is

(S10) $$\begin{array}{l} \langle \Delta {{\vec{R}}_{i}}^{2}\rangle =\frac{3k_{B}T}{\gamma }{\left(\Gamma^{-1}\right)}_{ii}\\ \langle \Delta {\vec{R}}_{i}\cdot \Delta {\vec{R}}_{j}\rangle =\frac{3k_{B}T}{\gamma }{\left(\Gamma^{-1}\right)}_{ij} \end{array}$$

Hence, the fluctuation of residue *i* is a constant times the diagonal element *ii* of the inverse Γ(the variance of *i*) ! We should note that the fastest motions that contribute the <Δ**R⃗***_i_*^2^> may not be well within the classical limit (*k_B_T* >>ħω). However, the inaccuracy of fast motions resulting from the quantum effects can be alleviated by the following facts: (1) The contribution of the fastest motions to the overall *size* of the fluctuations is extremely small; the fast modes are led by small *λ_k_*^−1^ (as explained in the equation 5 of the main text) (2) For CG models such as GNM, the fastest motions take place at the residue level and fastest C_α_ motions should be well above hundreds of femtoseconds (fs) or >1 picosecond (ps), which are still within the classical range, although the fastest motions on the residue level reported thus far, to the best of our knowledge, occurs at 130 cm^−1^ (or ~250 fs) as an ‘effective’ frequency for all the atoms in a residue (Moritsugu and Smith, 2007).

ReferencesArkhipovAFreddolinoPLSchultenK2006aStability and dynamics of virus capsids described by coarse-grained modelingStructure14176771716136710.1016/j.str.2006.10.003ArkhipovAFreddolinoPLImadaK2006bCoarse-grained molecular dynamics simulations of a rotating bacterial flagellumBiophys. J914589971699787110.1529/biophysj.106.093443PMC1779929BrooksBRBruccoleriREOlafsonBD1983CHARMM: A program for macromolecular energy, minimization and dynamics calculationsJ. Comp. Chem4187217ChuJWVothGA2007Coarse-grained free energy functions for studying protein conformational changes: a double-well network modelBiophys. J. BioFAST biophysj10711206010.1529/biophysj.107.112060PMC208424117704151IkeguchiMUenoJSatoM2005Protein structural change upon ligand binding: linear response theoryPhys Rev Lett94078102-141578385810.1103/PhysRevLett.94.078102JeffreyG1997An Introduction to Hydrogen Bonding New YorkOxford University PressMaragakisPKarplusM2005Large amplitude conformational change in proteins explored with a plastic network model: adenylate kinaseJ. Mol. Biol352807221613929910.1016/j.jmb.2005.07.031MartinetzTSchultenK1994Topology representing networksNeur Netw7507522MichelettiCCarloniPMaritanA2004Accurate and efficient description of protein vibrational dynamics: comparing molecular dynamics and Gaussian modelsProteins55635451510362710.1002/prot.20049MingDWallME2005Allostery in a coarse-grained model of protein dynamicsPhys. Rev. Lett9519810381638403010.1103/PhysRevLett.95.198103MiyashitaOOnuchicJNWolynesPG2003Nonlinear elasticity, proteinquakes, and the energy landscapes of functional transitions in proteinsProc. Natl. Acad. Sci. U.S.A1001257051456605210.1073/pnas.2135471100PMC240658MiyashitaOWolynesPGOnuchicJ2005Simple energy landscape model for the kinetics of functional transitions in proteinsJ. Phys. Chem. B1091959691685118010.1021/jp046736qMoritsuguKSmithJC2007Coarse-Grained Biomolecular Simulation with REACH: Realistic Extension Algorithm via Covariance HessianBiophys. J100346034691769346910.1529/biophysj.107.111898PMC2072085

## Figures and Tables

**Table 1:** Main features of CG-ENM.

EN Models	Nodes represent	Parameters in eq 1ζ	Matrix Dimension£	t_H_ or t_Γ_*
**GNM** (Bahar et al. 1997)	C_α_s	*E_0_* = *0,* γ = c*, w_G_* = *1, w_T_= 0, n* = *N, R_c_* = 7–15Å	*N* × *N*	1
**CNM** (Kondrashov et al. 2007)	C_α_s	γ = 1 for |i − j| = 1and 0.1 for |i − j| ≠ 1; E_0_ = 0, *w_G_* = 1, *w_T_* = 0, *n* = *N; R_c_* = 4 or 4.5Å (ab denotes atom *a* in *i* and atom *b* in *j* that are the closest atoms between *i* and *j*)	*N* × *N*	1
**ANM** (Atilgan et al. 2001)	C_α_s	E_0_ = 0, γ= c, *w_G_* = 0, *w_T_* = 1, *n* = *N, R_c_* = 10–15Å	3*N* × 3*N*	27
**HENM** (Hinsen 1998, 1999)	C_α_s	E_0_ = 0, *w_G_* = 0, *w_T_* = 1, *n* = *N*	3*N* × 3*N*	27
**βGM** (see Supplemental)	C_α_s, C_β_s	γ = 1 for C_α_–C_α_ and 0.5 for C_α_–C_β_and C_β_–C_β_; E_0_ = 0, *w_G_* = 0, *w_T_* = 1, *n* = *2N, , R_c_* = 7Å	3*N* × 3*N*	27
**BENM** (see Supplemental)	C_α_s	E_0_ = 0, *w_G_* = 0, *w_T_* = 1, *n* = *N*; γ and *R_c_* are obtained in minimizing KLD with atomistic Hessian	3*N* × 3*N*	27
**DNM** (Kondrashov et al. 2007)	C_α_s	E_0_ = 0, *w_G_* = 0, *w_T_* = 1, *n* = *N,* γ (**|r⃗***ij*^0^|) = 1/tr(H*_d_*), *d* = *R_c_* = 2.3, 3.3,5,7,9,11Å; *ab* denotes atom *a* in *i* and atom *b* in *j*	3*N* × 3*N*	27
**RTB/BNM** (Durand et al. 1994/Li and Cui, 2002)	blocks§	**H** from detailed potential	6*n*_B_ ×6*n*_B_	216
**Tirion’s** (Tirion, 1996)†	atoms	E_0_ = 0, γ= c, *w_G_* = 0, *w_T_* = 1, *n* = *N_a_, R_c_* = 5.9Å	3*N_a_* × 3*N_a_*	27000
**DWNM** (see Supplemental)	C_α_s	*w_G_* = 0, *w_T_* = 1, *n* = *N,* γ does not depend on (**|r⃗***ij*^0^|) but is a function of conformer *m*¶; *R_c_* = 13Å	N/A	N/A
**PNM** (see Supplemental)	atoms	*w_G_* = 0, *w_T_* = 1, *n* = *N_a_,* γ does not depend on (**|r⃗***ij*^0^|) but is a function of conformer *m*¶; *R_c_* = 4.5–9.5Å	N/A	N/A
**QEDM** (see Supplemental)	quantized nodes	E_0_ = 0, γ = c, *w_G_* = 0, *w_T_* = 1, *n* = *N_n_, R_c_* = 13Å	3*N_n_* × 3*N_n_*	27 (if *N_n_* = *N*)

$E=E_{o}+\sum_{i,j=1}^{n}\frac{\gamma (\left|{\vec{r}}_{ij}^{0}\right|)}{2}H(R_{c}-\left|{\vec{r}}_{ab}^{0}\right|)\left[w_{G}\left({\vec{r}}_{ij}-{\vec{r}}_{ij}^{0}\right)•\left({\vec{r}}_{ij}-{\vec{r}}_{ij}^{0}\right)+w_{T}{\left(\left|{\vec{r}}_{ij}\right|-\left|{\vec{r}}_{ij}^{0}\right|\right)}^{2}\right]$ (eq 1);

† Note that standard NMA has Hessian of the same size as Tirion’s hence same diagonalization time.

§ with user-defined number of atoms.

¶ when the structure is in the energy well of the conformer *m* at a given external parameter λ.

ζ c is constant; *ab* = *ij* if not stated otherwise; *i* and *j* denote residues if *n* = *N,* or atoms if *n* = *N_a_*; eq 1 is not applied for RTB.

£ the dimension of the square matrix **H** or **Γ**; *N* and *N_a_* is the number of residues and atoms respectively; *N* ≈ 10 *N_a_*; *n_B_* = *N* if 1 residue per block; *N_n_* is the number of quantized nodes.

* t_H_ and t_Γ_ are the time taken to diagonalize the **H** or **Γ** (all the modes) using the standard subroutine ; in relative unit as setting the time taken by GNM as unity.
